# Comment on Crettenand et al. Is Continuous Wound Infiltration a Better Option for Postoperative Pain Management after Open Nephrectomy Compared to Thoracic Epidural Analgesia? *J. Clin. Med.* 2023, *12*, 2974

**DOI:** 10.3390/jcm12185917

**Published:** 2023-09-12

**Authors:** Christian Marco Beilstein, Patrick Yves Wuethrich

**Affiliations:** Department of Anaesthesiology and Pain Medicine, Inselspital, Bern University Hospital, University of Bern, 3010 Bern, Switzerland; patrick.wuethrich@insel.ch

We have read with great interest the retrospective study recently published by Crettenand et al. in this journal [1]. We acknowledge the authors’ work but would like to add some comments and thoughts as their conclusions may be premature.

Overall pain control should be carefully planned in order to reduce early postoperative pain and also to prevent the onset of potential chronic pain syndromes. Nowadays, we have numerous analgesic options within ERAS programs. However, analgesia should not only result in lower pain scores (NRS or VAS), but also in better quality of recovery [2]. This can be easily assessed by a quality of recovery score like the QoR-15 or QoR-40. Unfortunately, this was not assessed in this study.

We wonder why another retrospective study is needed as numerous RCTs focusing on the topic of peripheral nerve block (e.g., paravertebral block, continuous surgical site analgesia) versus thoracic epidural analgesia (TEA) or opioid-based systemic analgesia have been published. The availability of valuable alternatives to TEA is not new per se [3,4].

TEA is still considered to be the gold standard, serving as a comparator for alternative techniques [5]. This is confirmed with this series. We disagree with the authors’ statement that patients with TEA had ‘slightly’ lower pain scores. Patients treated with TEA clearly had lower mean pain scores (corresponding to the classification of mild pain) compared to those subjected to continuous wound infiltration (CWI), which displayed moderate pain scores, at least within the first 24 h postoperatively. By definition, moderate pain ‘interferes with regular activities’, which is in disaccord with the fact that patients with CWI could be mobilized earlier compared to patients with TEA [6]. We therefore believe that patients with CWI were mobilized despite higher pain scores, an important bias in the interpretation of the benefit of CWI in terms of recovery, including reduced hypotension. At this point, the authors seem to misinterpret the conclusion of the original paper by Gramigni et al., stating that hemodynamic assessment does not predict an inability to walk after thoracic and abdominal surgery. Early mobilization should be tried irrespective of blood pressure or orthostatic changes in postoperative patients with TEA [7].

Acute severe pain in the immediate postoperative period is often considered to be related to the persistence of postsurgical pain after various procedures. Unfortunately, no information was provided if pain scores were assessed in rest or during coughing or mobilization. This is of importance, as sufficient analgesia is needed for mobilization to reduce postoperative complications. Looking at the pooled data of 3 series in our institution, we found median numeric rating scores of 0 [IQR 0 to 2] at rest and 2 [0–5] during mobilization [8,9]. Finally, a significant aspect of the quality of analgesia delivered using TEA is the visceral component provided by local anesthetic delivery directly to the neuraxis.

Patients with CWI received significantly more opioids during the first 3 days postoperatively, illustrating that CWI alone does not provide sufficient analgesia. Unfortunately, the quantity of opioids administered in both groups was not reported. A specification would be helpful as we only know that more than half of the patients with CWI needed the concomitant administration of a strong opioid. Also, as described in the methods, the dose of morphine hydrochloride administration was set to 0.1 mg/kg. However, the administration of morphine had to be adapted to renal function. As renal function is often impaired after renal surgery, there are other better options like methadone or hydromorphone. Finally, patients receiving increased doses of postoperative opioids were at higher risk for chronic use. This is of importance as around 11 to 26% of all open renal surgery patients will develop chronic pain postoperatively [10]. Furthermore, the clearance of local anesthetics is impaired in severe renal dysfunction, and acidosis as well as uremia increase the concentration of free local anesthetics. Compared to fascial plane blocks, the risk of systemic toxicity is considered to be lower for the epidural administration of local anesthetics, especially when combined with epidural epinephrine, resulting in decreased plasma levels for free local anesthetics [11,12]. Unfortunately, the authors gave us no information about postoperative renal function, and so no conclusions could be drawn from this series in this regard.

Finally, the earlier removal of CWI was only carried out due to the design of the protocol, as the elastomeric pump was programmed to deliver ropivacaine for 72 h. Hence, the statement that adequate oral pain control was achieved earlier for CWI reflects the study design rather than its superiority. However, we acknowledge that the removal of TEA and the switch to oral analgesic treatment remains challenging and is often neglected. We recently showed that the administration of oral opioids to reduce and remove TEA is of no advantage compared to the use of placebo [13]. In the time of multimodal anesthesia, when aiming for the reduced perioperative administration of opioids and opioid-free anesthetic concepts, we believe that TEA has a role to play in major open surgery [14,15].

We were surprised to read that CWI resulted in a 40% overall cost reduction, being even more shocked to discover that there were no data or details supporting that statement in the paper. The total time in the operating room (OR) is similar in both groups (TEA, 282 min vs. CWI, 279 min), despite the longer induction time in the TEA group (12 min). The author’s statement in the discussion, that the 40% cost reduction is due to reduced OR time, is therefore not supported by the data provided. Knowing the overall cost of one minute OR time in our tertiary institution (from the same country), a difference of 3 min in OR time (but a trend towards a higher surgical time) will not result in significant cost savings. Even taking into consideration an additional daily visit by a trained analgesia nurse and the cost of consumables (for both groups), neither an additional half-day hospitalization nor reduced nursing workload is likely to result in a difference of CHF 11,200 per patient. We wonder if the use of other, unreported resources, such as intermediate care or prolonged post-anesthetic care stays, could be an issue. Therefore, a detailed description of generated costs would be helpful.

As correctly stated by the authors as a limitation, they included a low number of patients in the study but also needed a long observation period of nearly 10 years. This introduces important biases such as different surgeons and the development (ideally improvement) of surgical and perioperative techniques over a decade. It also needs to be clarified whether TEA and CWI were equally distributed over the observation time.

From our point of view, CWI is a possible alternative to TEA for patients with contraindications or for patients at risk for hypotension, but at the expense of higher early postoperative opioid consumption and poorer analgesia. Still, epidural analgesia provides superior pain control and potentially offers benefits including early mobility, improved cardiopulmonary function and reduced risk of venous thromboembolism [16,17]. The evaluation of treatment options continues to be difficult for the clinician and the need for larger, prospective, and well-designed trials covering all dimensions required for treatment decisions and specific patient groups remains.
